# Sarcoma

**Published:** 1890-02

**Authors:** R. C. Mason


### SARCOMA.

By Dr. R. C. Mason.

The subject was a sorrel mare 8 years old, 14 hands high, weighing in ordinary flesh about 900 pounds. She was considered extra as a worker, as she worked against two other horses on a farm of 160 acres, and kept in good condition until about two months before her death, when she began to go off her feed and fail. After resting for several weeks, and rapidly failing, the owner brought her to my Infirmary November 9th, 1889. When she arrived she was suffering and very weak from super-purgation, the effect of salts which had been given a day or two previously, and the exercise of traveling from her home. She yielded to treatment, and on the 10th had a pulse 72, and a temperature of 104°, the respiration was slightly abdominal and hurried; on percussion found slight dullness on the lower part of right lung. The abdomen was enlarged; her appetite was capricious and very poor, and she lost flesh rapidly. There was no change in visible mucous membrane. The fæces were dark and scanty. The urine was apparently normal.

Her temperature varied from day to day, and would vary in two hours from 99½° to 104°, or the reverse, with a pulse from 44 to 80. No medicine had the slightest perceptible effect. She failed rapidly, and died on the 25th.

*Post-mortem* revealed a liver seventy-three pounds in weight. There were attached to the floating colon, near its commencement, three abnormal growths, oval in shape, about 6 × 10 inches. These averaged fifteen pounds apiece. They had no connection with the interior of bowel and were attached by a small pedicle. Their covering, colored about like the intestines, was on the average one-quarter inch thick, and in places ossified. Two contained a coffee-colored fluid with thick sediment; the third a little; the largest no fluid, but was filled with nodular growths varying in size from grapes to walnuts. The peritoneum was thickly studded with abnormal growths, single and in clusters, varying in size from peas to hens' eggs; these growths were also attached to the walls of the abdominal cavity; the thoracic cavity and its contents were normal. Kidneys normal.

[Ed. Note.—A large mass of the liver sent for inspection showed a magnificent specimen farcied with secondary (metastatic) sarcomas.]

